# PReS-FINAL-2233: Retrospective analysis of different treatment strategies in chronic non-bacterial osteomyelitis

**DOI:** 10.1186/1546-0096-11-S2-P223

**Published:** 2013-12-05

**Authors:** MM Kostik, IA Chikova, MF Dubko, LS Snegireva, VV Masalova, OV Kalashnikova, AY Mushkin, VG Chasnyk

**Affiliations:** 1 Hospital Pediatry, Saint-Petersburg State Pediatric Medical University, Russian Federation; 2 Non-pulmonary tb department, Scientific and research institute of physiopulmonology, Saint-Petersburg, Russian Federation

## Introduction

Pediatric chronic nonbacterial osteomyelitis (CNO) is a sterile inflammatory bone disorder in which innate and adaptive immunity dysfunction involved. Unifocal and multifocal disease courses are known. The modern treatment modalities include non-steroid anti-inflammatory drugs (NSAIDs), steroids, sulfasalazine (SSZ), methotrexate (MTX), bisphosphonates and biologic drugs - TNFα and IL1β-antagonists, with limited data.

## Objectives

The aim of our study was to assess children with CNO and to evaluate efficacy of treatment modalities.

## Methods

Our cohort of CNO patients included 22 children, 8 boys and 14 girls. Monofocal disease course was in 9/22 children (40.9), multifocal in 13/22 (59.1) with mean 6 foci per patient. Histological confirmation was made in 13/22. Repeated MRI, CT and bone scintigraphy was performed in all patients. 3 patients have family history of autoimmunity (1 Crohn's disease, 1 - psoriasis, 1 - ankylosing. 16 patients (72,7%) had comorbid autoimmune diseases (different types of JIA): 5 had monoarthritis, 1 arthritis with uveitis, 1 - psoriatic arthritis, 1 - polyarthritis PF neg, 6 had enthesitis-related arthritis (3 had ankylosing spondyloarthritis) and 1 had Crohn's disease. Spine involvement was in 5/22 (22.7). Onset age was 8.5 (6.3; 10.5) years, the right diagnosis delay was 3.6 (1.7; 9.5) months.

## Results

Fever at onset, high painVAS and parental VAS scores highly correlated with risk of relapse disease course. Treatment: effectiveness of NSAID only 3/10 (30%), SSZ - 1/5 (20%), corticosteroids - 0/3 (short-term effect only), MTX - 4/7 (57.1%), pamidronate (PAM) with partial response 2/12 (16.7%) and with complete response - 10/12 (83.3%). Biologics - adalimumab and etanercept were effective in 3/4 (75%) patients, who fail to NSAID, MTX, PAM and SSZ. During disease course treatment lead to decreasing sings of disease activity, such as: parental VAS (p = 0.015), pain VAS (p = 0.026), MDVAS (p = 0.026), CRP (p = 0.0008), WBC (p = 0.006), ESR (p = 0.00024), PLT (0.014). The main effectiveness belonged to PAM (p = 0.003) and biologics (p = 0.07) in decreasing of pain VAS (-100% and -80%), parental VAS (-92% and -74%) and MD VAS (-93% and -70%, respectively). We calculated the cumulative probability of survival (event of interest: CNO flare) in the entire patient sample, depending the kind of treatment (PAM, MTX and NSAID) obtained by the Kaplan-Meier method. Significant difference was proved comparing 3 therapeutical branches (p = 0.028). MTX treatment was effective (p = 0.04), as well as PAM (p = 0.01) than NSAID. Only flu-like syndrome during PAM treatment was in 10/12 (83.3%). No any others side effects were reported. All patients who had flu-like syndrome on first infusion had complete response to PAM, vice verse patients, who had no such complication had only partial response to this treatment.

## Conclusion

CNO is a group of chronic inflammatory conditions associated with different rheumatic diseases. The most effective treatment modalities were PAM, biologics and MTX. PAM was safety and can reach the rapid response and maintain long sustained remission.

## Disclosure of interest

None declared.

